# The evolving role of liver transplantation for metastatic colorectal cancer: current perspectives and future directions

**DOI:** 10.3389/fsurg.2025.1608467

**Published:** 2025-07-17

**Authors:** Victoria A. Bendersky, Danae G. Olaso, Gabriel T. Schnickel

**Affiliations:** Department of Surgery, Division of Transplant Surgery, University of California San Diego, San Diego, CA, United States

**Keywords:** metastatic colorectal cancer, hepatic metastasis, liver transplantation, MELD exception, machine perfusion

## Abstract

Liver transplantation is increasingly being explored as a treatment option for select patients with metastatic colorectal cancer (mCRC). Historically, transplantation for mCRC was abandoned due to poor long-term outcomes and high recurrence rates. However, recent advancements in patient selection, immunosuppressive strategies, and donor organ availability have led to a renewed interest in this approach. Studies have demonstrated that highly selected patients undergoing liver transplantation can achieve significantly improved survival rates compared to those receiving standard systemic therapies. The implementation of Model for End-Stage Liver Disease (MELD) exception points, improved donor preservation techniques such as machine perfusion, and the growing role of living donor liver transplantation have further supported its feasibility. As research continues, liver transplantation may emerge as a crucial component of a multidisciplinary strategy for treating colorectal liver metastases, offering a select group of patients a chance at prolonged survival and improved quality of life.

## 1 Introduction

Metastatic colorectal cancer (mCRC) is a significant health concern globally. Colorectal cancer (CRC) is the third most common cancer worldwide, with approximately 1.85 million new cases and 850,000 deaths annually. In the United States, there are nearly 150,000 new cases diagnosed and over 50,000 deaths each year due to CRC (1, 2). Nearly half of patients with colorectal cancer will develop metastatic disease (3). Additionally, 25%–30% of patients present with liver metastases at the time of diagnosis (synchronous liver metastases). Another 20%–25% of patients will develop liver metastases metachronously, or after the initial diagnosis and treatment of the primary tumor (4).

**Table 1:** Examination of modern studies from Europe, Canada, and the US.

Study	Year	Design	Inclusion criteria	Exclusion criteria	Size	Results
Hagness et al. (SECA-I) (15)	2013	Prospective	Liver-only CLMs, excised primary tumors, and at least 6 weeks of chemotherapy	Extrahepatic metastases, other malignancy	*N* = 21	OS rate at 1, 3, and 5 years were 95%, 68%, and 60%, DFS was 35% at 1-year
Toso et al. (47)	2017	Retrospective	Primary tumor resected, partial response to chemotherapy	Extrahepatic metastases, other malignancy	*N* = 12	OS rates at 1, 3, and 5 years: 83%, 62%, 50%; DFS rates at 1, 3, and 5 years: 56%, 38%, 38%
Dueland et al. (SECA-II) (14)	2020	Prospective	Excised primary tumor <10 cm, nonresectable liver-only metastases determined by CT/MRI/PET, at least 10% response to CTX, time from diagnosis to liver transplant >1 year	Extrahepatic metastases, other malignancy	*N* = 15	OS at 1, 3, and 5 years were 100%, 83%, and 83%; DFS at 1, 2, and 3 years were 53%, 44%, and 35%; OS from time of relapse at 1, 2, and 4 years were 100%, 73%, and 73%
Smedman et al. (SECA-II D Arm) (36)	2020	Prospective, patients who were excluded from SECA-II arms A, B or C	Same as SECA-II, allowed for patient with resectable pulmonary metastases, ECD organ donor	Extrahepatic metastasis (other than pulmonary), BMI above 30 kg/m2 and liver metastases larger than 10 cm	*N* = 10	DFS was 4 months, OS was 18 months
Hernandez-Alejandro et al. (25)	2022	retrospective, US and Canada centers	primAry tumor resected, response to chemotherapy 6–12 months, no evidence of extrahepatic disease, CEA < 80 ng/dl, no synergistic tumor mutations		*N* = 10	RFS was 62% and OS was 100% at 1.5 years
Sasaki et al. (24)	2023	Retrospective	No specific inclusion criteria		64 listed, 46 transplanted	LDLT 26 patients, DDLT 20 patients; DFS at 1-, and 3-years were 75.1%, and 53.7%, OS at 1- and 3-years were 89.0% and 60.4%
Rajendran et al. (23)	2023	Prospective, transplanted vs. resected vs. control (CTX alone) in LDLT in Canada	Primary tumor resected, time from resection to transplant ≥6 months, CTX ≥ 3 months, CEA stable or decreasing	Extrahepatic metastases, other malignancy, prior lung resection, progression of metastases, *BRAF* tumors	81 total, 7 transplant, 22 resection, 48 controls	Recurrence-free survival was superior in the LDLT group (1-year 85.7% vs. 11.4%; 3-year 68.6% vs. 11.4%; no difference in OS between transplanted and resected groups
Kaltenmeier et al. (48)	2023	Retrospective; largest single center analysis of patients undergoing LDLT for mCRC	Resected primary tumor, 6–12 wks of CTX, CEA < 100 ng/dl	Extrahepatic metastases, other malignancy	*N* = 10	Mean RFS was 2.2 years, Mean OS was 3.0 years
Solheim et al. (49)	2023	Retrospective, long term follow up of SECA-I patients	Resected primary tumor, at least 6 weeks of CTX	Extrahepatic metastases, other malignancy	*N* = 23	5-year and 10-year OS after LT were 43.5% and 26.1%; For patients with Oslo Score of 0 or 1, the 5-year and 10-year actual OS was 75% and 50%, respectively (*n* = 6). For patients with Oslo Score of 2, the 5-year and 10- year actual OS 50% was 33% (*n* = 6). All patients with Oslo score 3 or 4 were deceased 86 months post-LT (*n* = 9)
Adam et al. (TRANSMET) (22)	2024	Multi-center prospective RCT, randomized to CTX vs. CTX + LT	Resected primary tumor, >3 months CTX with response to CTX	Extrahepatic metastases, *BRAF* mutated primary tumor	*N* = 94	5-year overall survival was 56·6% for LT + CTX and 12·6% for CTX alone; Median PFS was 17.4 mos vs. 6.4 mos; Fifteen (40%) patients were disease-free

The incidence of colorectal cancer has changed dramatically since the introduction of early screening techniques in the 1990s. Approximately 10%–12% of new CRC diagnoses occur in patients younger than 50 years of age (5). The increased incidence of early onset (individuals <50 years of age) CRC is not yet well understood. However, there are multiple studies that found early onset CRC were associated with later stage distal colon or rectal tumors, poorly differentiated tumors, or mucinous and signet ring tumors that are associated with poor outcomes (6, 7). With the expanding role of liver transplantation in the treatment of mCRC, more patients with mCRC may benefit from liver transplantation.

### 1.1 Overview of metastatic colorectal cancer: current treatment options and limitations

The prognosis of mCRC remains poor, with a 5-year relative overall survival rate of approximately 15%. Advances in treatment, including systemic therapies and targeted treatments based on molecular profiling, have improved survival outcomes, but cures remain uncommon (1, 2). The current treatment options for colorectal cancer with metastasis to the liver include surgical resection, systemic therapy, and various liver-directed therapies.

Surgical resection remains the only potentially curative treatment for liver-limited metastatic colorectal cancer. Approximately 20%–30% of patients with colorectal cancer with liver metastasis are candidates for surgical resection, which can significantly improve survival outcomes. The American Society of Clinical Oncology (ASCO) recommends neoadjuvant chemotherapy followed by surgery vs. surgery alone for patients who are candidates for potentially curative resection of liver metastases (2).

Systemic therapy is essential for patients with unresectable liver metastases or those with extrahepatic disease. The National Comprehensive Cancer Network (NCCN) guidelines recommend systemic chemotherapy regimens such as FOLFOX (fluorouracil, leucovorin, and oxaliplatin), FOLFIRI (fluorouracil, leucovorin, and irinotecan), or FOLFOXIRI (fluorouracil, leucovorin, oxaliplatin, and irinotecan), often combined with biologic agents like bevacizumab or cetuximab, depending on the molecular profile of the tumor. Pembrolizumab is recommended for patients with microsatellite instability-high or deficient mismatch repair tumors (2, 8).

Liver-directed therapies include ablation techniques (radiofrequency ablation, microwave ablation), transarterial chemoembolization (TACE), selective internal radiation therapy (SIRT), and hepatic arterial infusion (HAI) chemotherapy. These therapies are particularly useful for patients with unresectable liver-confined disease or those who are not candidates for surgery due to comorbidities. The American College of Radiology (ACR) also supports the use of these interventional radiological techniques to address limitations of curative resection (9–12).

Limitations of these treatments include the risk of postoperative liver failure due to insufficient future liver remnant volume, the potential for disease relapse after liver surgery, and the adverse effects associated with systemic and liver-directed therapies. Additionally, the role of stereotactic body radiation therapy (SBRT) and liver transplantation in the management of colorectal cancer with liver metastasis are still being evaluated, with ongoing studies needed to establish definitive recommendations (9, 10, 13).

### 1.2 Liver transplantation as an alternative treatment approach

The rationale for exploring liver transplantation as a treatment option for metastatic colorectal cancer, specifically in the context of colorectal cancer with metastasis to the liver, is based on several key factors: improved survival outcomes, patient selection, and management of recurrence.

Liver transplantation has demonstrated significantly improved overall survival rates in highly selected patients with nonresectable liver-only metastases. Prospective studies have shown 5-year survival rates of up to 80% in selected patients, which is markedly higher than the approximately 10% 5-year survival seen with palliative chemotherapy alone (14–17).

Advances in patient selection criteria have been crucial. Factors such as carcinoembryonic antigen (CEA) levels, response to chemotherapy, size of liver lesions, and time from primary tumor resection to transplantation have been identified as important prognostic indicators. These criteria help identify patients who are more likely to benefit from liver transplantation (14, 17).

Although recurrence is common, it often occurs as slow-growing metastases, particularly in the lungs, which can be amenable to further curative interventions. This contrasts with the rapid progression typically seen in patients treated with chemotherapy alone (16, 18). The Fong clinical risk score is a preoperative scoring system that was also developed to predict recurrence after hepatic resection for mCRC. Five variables included are nodal-positive primary, disease free interval <12 months, >1 tumor, preoperative CEA > 200 ng/ml, and tumor size >5 cm with one point assigned per criterion (19). The total score was highly predictive of long-term outcomes with scores up to two having a more favorable outcome.

## 2 Evolution of standard of care and the historical transplant experience

Liver transplantation for colorectal cancer metastasized to the liver has undergone significant evolution, marked by key historical outcomes and advancements in patient selection and perioperative management. Initially, liver transplantation for colorectal cancer with liver metastasis was abandoned in the 1990s due to poor outcomes and high rates of recurrence, with 5-year overall survival rates as low as 18% (15). Liver transplantation for mCRC re-emerged after the landmark SECA I and SECA II studies from Norway. Both studies focused on a highly selective patient population with CRC and unresectable liver-limited metastases. The SECA-I study published in 2013 found that patients who underwent primary tumor resection and at least 6 months of chemotherapy followed by liver transplant demonstrated a 5-year overall survival of 60% (15). This study highlighted the importance of stringent selection criteria, including liver-only metastases, excised primary tumors, and response to chemotherapy.

Subsequently, the SECA-II study further refined these criteria and reported even better outcomes. In this study, patients with CRC and unresectable liver metastases on imaging with favorable prognostic factors, such as low carcinoembryonic antigen (CEA) levels and significant response to chemotherapy, had a 5-year overall survival rate of 83% in patients (14). These findings underscored the potential for liver transplantation to offer long-term survival benefits comparable to other indications for liver transplantation.

A prospective cohort study at Oslo University Hospital evaluated multiple clinical trials on liver transplant for colorectal liver metastases to determine disease recurrence, overall survival and survival after relapse in the modern age. This led to the development of the Oslo scoring system which identified negative predictive factors for overall survival including tumor size greater than 5.5 cm, disease progression on chemotherapy, plasma CEA > 80 μg/L, and time interval between primary tumor resection and liver transplant of less than 2 years (16). Patients with Oslo score of 0 had an overall 5- and 10-year survival of >80% while those with Oslo score of 0–2 had a median 5- and 10-year OS rate of 63% and 45% (16).

A systematic review and meta-analysis by Varley et al. reported pooled 5-year overall survival rates ranging from 50% to 80%, with disease-free survival (DFS) rates between 35% and 56% (20). Another meta-analysis by Dawood et al. confirmed these findings, showing a pooled 5-year overall survival of 53% and highlighting the common recurrence pattern of slow-growing pulmonary metastases (21).

Overall, the historical outcomes of liver transplantation for colorectal cancer with liver metastasis have improved significantly due to better patient selection and perioperative management, making it a viable treatment option for selected patients with nonresectable liver-only metastases.

## 3 Current literature review: examining recent data from Europe, Canada, and the US

Recent studies from Europe, Canada, and the United States have provided compelling evidence supporting the use of liver transplantation as a treatment for metastatic colorectal cancer in highly selected patients (Table 1).

A multicenter, randomized controlled trial from Europe, the TransMet study, demonstrated that liver transplantation plus chemotherapy significantly improved 5-year overall survival to 56.6% compared to 12.6% with chemotherapy alone in patients with permanently unresectable colorectal cancer with liver metastasis (22). This study underscores the potential of liver transplantation to offer a survival benefit in this patient population.

In Canada, the Toronto Living Donor Liver Transplant Program reported promising outcomes with living donor liver transplantation (LDLT) for unresectable colorectal cancer with liver metastasis. This prospective study recruited patients with unresectable CRLM (defined as bilobar disease) receiving systemic chemotherapy. The participants were then divided into three groups: patients who underwent LDLT, patients who underwent chemotherapy alone, and patients who underwent chemotherapy, converted to resectable disease, and underwent liver resection only. The study found that LDLT provided superior recurrence-free survival compared to resection, with a 3-year recurrence-free survival of 68.6% vs. 11.4% (23). This highlights the potential of LDLT as a viable option for selected patients.

In the United States, a systematic review and meta-analysis by Dawood et al. reported pooled 5-year overall survival rates of 53% for liver transplantation in patients with nonresectable colorectal cancer with liver metastasis, with the lungs being the most common site of recurrence (21). This meta-analysis supports the notion that liver transplantation can offer substantial survival benefits.

Sasaki et al. identified 46 patients with mCRC who underwent liver transplant in the US. 21% of patients experienced disease recurrence. 1- and 3-year disease free survival rates were 75% and 53%, and 1- and 3-year overall survival rates were 89% and 60%, respectively (24). Hernandez-Alejandro et al. identified 10 patients with mCRC who underwent LDLT. 3 patients had recurrence, disease free survival and overall survival rates were 62% and 100%, respectively, with a median follow up period of 1.5 years (25). While recent studies have a small cohort, they highlight the improvement in patient outcomes and need for stringent selection criteria. In 2021, the International Hepato-Pancreato-Biliary Association published consensus guidelines to identify and select patients with non-resectable mCRC who benefit from liver transplantation (26). These guidelines are standardized nomenclature focused on patient selection, with strict clinic-patho-radiological criteria, evaluation of biological behavior, graft selection and organ allocation, and recipient immunosuppression and prevention or management of recurrent disease.

## 4 *BRAF*, *RAS*, and other genetic mutations

CRC primary tumors in the left colon have better outcomes than primary right colon tumors. This is because *BRAF* mutations are more likely to originate from the right colon and sporadic mutations from the left colon (27–30). *BRAF* mutations occur in about 10% of CRC and patients with CRC from *BRAF* mutations have increased risk of recurrence. Multiple retrospective studies found that patients with *BRAF* mutations, who underwent curative-intent resection with hepatectomy, were a predictor of worse overall survival and recurrence free survival (27, 29–33). In addition, those with *BRAF* mutations lack response to current anti-EGFR systemic chemotherapy treatments. Thus, patients with V600E *BRAF* mutations are not considered candidates for liver transplantation due to poor prognosis, risk of recurrence, and the decreased benefit of transplant. RAS mutations occur in about 50% of colorectal tumors, and while it is a negative prognostic factor, is not an absolute contraindication to liver transplantation.

Mismatch repair deficiency in mCRC occurs in about 5% of patients and results in high mutation burden and microsatellite instability (MSI-H). The KEYNOTE-177 study demonstrated that Pembrolizumab, a PD-1 blockade immunotherapy, had superior progression free survival and no difference in overall survival in patients with MSI-H mCRC (34). However, liver transplantation in patients with prior immune check point inhibitor therapy remains in flux. Multiple studies in patients with HCC who underwent immune check point inhibitor therapy prior to liver transplantation experienced an acute allograft rejection rate of 16%–37% (34, 35). Of note, patients with MSI-H and *BRAF* mutations mCRC tumors are contraindicated and often excluded in the current studies.

The follow up study for patients with extended criteria, the SECA-II arm D study, included patients with poorly differentiated or signet ring cell primary tumors as well as patients with primary tumors in the ascending colon and patients with extensive liver tumor burden. Median disease-free survival was 4 months, with the most common recurrence being pulmonary metastasis, and median OS was 18 months which was significantly lower compared to patients in the SECA-I and SECA-II trials (36). Thus, poorly differentiated mCRC tumors are contraindication to liver transplantation.

Overall, recent and current studies in mCRC and liver metastases aim to refine strict patient selection criteria for candidates of liver transplant to improve outcomes.

### 4.1 Future directions in liver transplantation for mCRC: MELD exception points

Model for End-Stage Liver Disease (MELD) exception points are used to prioritize patients with metastatic colorectal cancer for liver transplantation by assigning them a higher allocation MELD (aMELD) score. This is done to reflect the patient's increased risk of waitlist dropout and to ensure equitable access to transplantation.

Patients with colorectal cancer with liver metastasis often have a lower laboratory MELD (lMELD) score, which may not accurately represent their urgency for transplantation. MELD exception points are granted to these patients to adjust their priority on the transplant waitlist, thereby increasing their chances of receiving a liver transplant. This approach is supported by studies showing that patients with non-HCC MELD exceptions, including those with colorectal cancer with liver metastasis, have a higher likelihood of undergoing liver transplantation and a reduced risk of waitlist dropout when adjusted for aMELD (37, 38).

The allocation of MELD exception points are all adjudicated via the National Liver Transplant Oncology Review Board and United Network for Organ Sharing (UNOS). These boards evaluate the medical urgency and potential benefit of transplantation for each patient, ensuring that those with colorectal cancer with liver metastasis who meet specific criteria receive appropriate prioritization (37, 39).

In June 2024, the OPTN and UNOS approved updates to the transplant oncology allocation policy to include MELD exception points for patients with mCRC with unresectable liver metastases. Criteria for MELD exception points include resection of primary tumor with negative margins, no extrahepatic metastatic disease or local recurrence and stable disease on systemic chemotherapy. Exclusion criteria include extrahepatic disease after primary tumor resection, local relapse of primary disease, or CEA > 80 μg/L. A table summarizing these inclusion and exclusion criteria is included as Table 2 (40).

**Table 2:** MELD exception points: colorectal liver metastases.

Criteria type	Inclusion criteria	Exclusion criteria
Primary tumor	- Resected colorectal primary tumor (R0 resection)	- Unresected primary tumor - Rectal tumors with pelvic spread
Metastatic disease	- Liver-only metastases - <10 lesions (some protocols allow >10 if <10 cm cumulative) - No extrahepatic disease on PET/CT/MRI - Stable disease ≥6 months with chemotherapy	- Extrahepatic metastases (e.g., lung, bone) - Progressive disease on chemotherapy - Peritoneal carcinomatosis
Response to chemotherapy	- Partial or complete response to at least 6 months of systemic chemotherapy (e.g., FOLFOX, FOLFIRI) - CEA trend downward and preferably <80 ng/ml	- No response or progression on chemotherapy - CEA >100–200 ng/ml and rising
Time from diagnosis	- ≥12–24 months since diagnosis of metastatic disease - Sustained disease control	- <12 months since metastasis - Rapid progression within short time
Surgical feasibility	- Complete hepatectomy technically feasible - Performance status ECOG 0–1	- Incomplete resectability - Poor performance status (ECOG ≥2)
Other requirements	- Multidisciplinary tumor board approval - Enrolled in research protocol or trial - Informed consent for experimental nature	- Not eligible or willing to participate in trial - Transplant center does not offer protocol

In summary, MELD exception points are used to prioritize patients with metastatic colorectal cancer for liver transplantation by assigning them a higher MELD score, reflecting their increased risk and ensuring equitable access to transplantation.

The study by Sjule et al. utilized a discrete event simulation model to explore the effects of expanding liver transplantation eligibility to include CRLM patients in Norway (41). The model predicted that for every additional CRLM patient listed per year, the overall median wait-list time increased by 8% to 11%. Adding two additional CRLM patients under restrictive eligibility criteria increased the CRLM patients’ average life expectancy by 10.64 years and resulted in a net gain of 149.61 life-years over a 10-year period. This suggests a modest but significant increase in the number of liver transplants performed annually for CRLM patients. The study by Ueberroth et al. highlighted advances in donor organ preservation, including machine perfusion technology, have expanded organ availability, allowing for the inclusion of CRLM patients in liver transplantation programs (42). This expansion is supported by data showing comparable overall survival for liver transplantation in CRLM patients to other liver transplantation indications.

Additionally, the study by Adam et al. in the TransMet trial demonstrated that liver transplantation plus chemotherapy significantly improved 5-year overall survival compared to chemotherapy alone, supporting the validation of liver transplantation as a new standard option for patients with permanently unresectable liver-only metastases (22). This trial's findings suggest that incorporating MELD exception points and utilizing marginal livers through machine perfusion technology could further increase the number of liver transplants performed annually for CRLM patients.

### 4.2 Utilizing marginal or historically discarded livers with machine perfusion technology

Marginal or historically discarded livers can be utilized for liver transplantation in patients with metastatic colorectal cancer through the application of machine perfusion technology, specifically normothermic machine perfusion (NMP). NMP allows for the ex vivo preservation and functional assessment of livers that would otherwise be discarded due to concerns about their viability. This technology maintains the liver at physiological temperatures, providing oxygen and nutrients, which helps to reduce ischemia-reperfusion injury and allows for real-time evaluation of liver function.

Studies have demonstrated that NMP can successfully rescue a considerable proportion of marginal livers. For instance, the RESTORE trial reported that 72.7% of declined livers treated with NMP were successfully transplanted, with no graft-related deaths or primary nonfunction (43). Similarly, another study found that 71.5% of discarded livers subjected to NMP were deemed suitable for transplantation, with good post-transplant outcomes (44).

NMP has expanded the donor pool, increased the utilization of marginal allografts, and has been increasingly adopted in transplant centers across the US. UNOS reported the use of NMP rose to over 11% in 2022 compared to 3.5% in 2021 (45). The use of NMP can be particularly beneficial for colorectal cancer with liver metastasis patients, who often have lower MELD scores and may face longer wait times for transplantation. By expanding the donor pool with marginal livers, NMP can increase the availability of suitable grafts for these patients, potentially improving their survival outcomes (43, 44, 46). The adoption of NMP and other novel perfusion techniques is an important tool for increasing the number of liver allografts available as the indications for liver transplantation in the setting of transplant oncology continue to evolve.

## 5 Conclusion

Liver transplantation is emerging as a viable treatment option for select patients with metastatic colorectal cancer. Studies from Europe, Canada, and the United States demonstrate that liver transplantation can achieve 5-year survival rates exceeding those of standard systemic therapies, particularly when strict eligibility criteria are applied. The implementation of MELD exception points, advances in donor organ preservation through machine perfusion technology, and the growing acceptance of living donor liver transplantation further support the feasibility of expanding liver transplantation for colorectal liver metastases. However, ongoing research is needed to refine patient selection criteria, optimize immunosuppressive strategies, and address long-term outcomes, including disease recurrence. As the field continues to evolve, liver transplantation is likely to become an integral component of the multidisciplinary approach to managing metastatic colorectal cancer, offering select patients a potential for long-term survival and improved quality of life.
